# Autophagy Requirements for Eye Lens Differentiation and Transparency

**DOI:** 10.3390/cells12030475

**Published:** 2023-02-01

**Authors:** Lisa Brennan, M. Joseph Costello, J. Fielding Hejtmancik, A. Sue Menko, S. Amer Riazuddin, Alan Shiels, Marc Kantorow

**Affiliations:** 1 Department of Biomedical Science, Schmidt College of Medicine, Florida Atlantic University, Boca Raton, FL 33460, USA; 2 Department of Cell Biology and Physiology, University of North Carolina, Chapel Hill, NC 27599, USA; 3 Ophthalmic Genetics and Visual Function Branch, National Eye Institute, National Institutes of Health, Bethesda, MD 20892, USA; 4 Department of Pathology, Anatomy and Cell Biology, Sidney Kimmel Medical College, Thomas Jefferson University, Philadelphia, PA 19107, USA; 5 Department of Ophthalmology, Sidney Kimmel Medical College, Thomas Jefferson University, Philadelphia, PA 19107, USA; 6 The Wilmer Eye Institute, Johns Hopkins University School of Medicine, Baltimore, MD 21287, USA; 7 Department of Ophthalmology and Visual Sciences, Washington University School of Medicine, St. Louis, MO 63110, USA

**Keywords:** autophagy, lens, differentiation, cataract

## Abstract

Recent evidence points to autophagy as an essential cellular requirement for achieving the mature structure, homeostasis, and transparency of the lens. Collective evidence from multiple laboratories using chick, mouse, primate, and human model systems provides evidence that classic autophagy structures, ranging from double-membrane autophagosomes to single-membrane autolysosomes, are found throughout the lens in both undifferentiated lens epithelial cells and maturing lens fiber cells. Recently, key autophagy signaling pathways have been identified to initiate critical steps in the lens differentiation program, including the elimination of organelles to form the core lens organelle-free zone. Other recent studies using ex vivo lens culture demonstrate that the low oxygen environment of the lens drives HIF1a-induced autophagy via upregulation of essential mitophagy components to direct the specific elimination of the mitochondria, endoplasmic reticulum, and Golgi apparatus during lens fiber cell differentiation. Pioneering studies on the structural requirements for the elimination of nuclei during lens differentiation reveal the presence of an entirely novel structure associated with degrading lens nuclei termed the nuclear excisosome. Considerable evidence also indicates that autophagy is a requirement for lens homeostasis, differentiation, and transparency, since the mutation of key autophagy proteins results in human cataract formation.

## 1. Introduction

The lens is a unique encapsulated tissue comprised of an anterior layer of cuboidal epithelial cells overlying a core of elongated and transparent fiber cells [1,2,3]. The precise coordination of multiple signaling, transcriptional, and translational pathways is essential for the differentiation of immature lens epithelial cells into elongated, organelle-free, and transparent lens fiber cells. The differentiation of lens epithelial cells into lens fiber cells is continuous throughout life. Consistent with this, disruption of the lens differentiation program during development or later in life results in the formation of cataract, which, despite advances in cataract surgery, remains a leading cause of visual disability worldwide [4,5]. 

**Figure 1:**
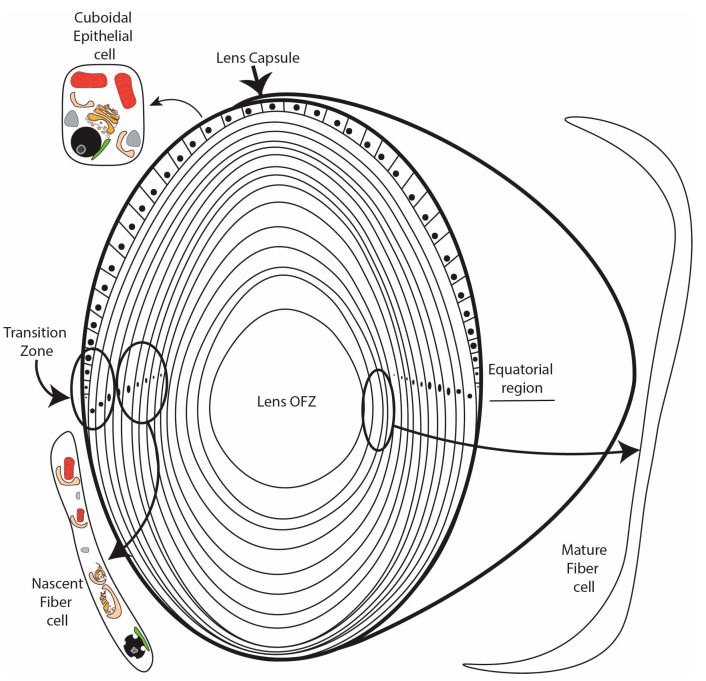
Anterior to posterior (sagittal) schematic of the eye lens. The lens is bound by a collagenous capsule. An anterior layer of largely quiescent cuboidal epithelial cells overlies a core of elongated protein-packed organelle-free fiber cells. Epithelial cells at the lens equator/transition zone exit the cell cycle, detach from the capsule, turn, and begin to elongate. This process of continuous differentiation adds layer after concentric layer onto the lens. Nascent fiber cells begin to elongate and show the first signs of autophagy-driven organelle degradation. Fully mature fiber cells, which are devoid of organelles, comprise the core of the lens in the region known as the organelle-free zone (OFZ).

The lens differentiation program is initiated in epithelial cells located at the lens equator (Figure 1). Upon activation by FGF [6,7,8], IGF [9,10], and possibly other inductive signals, these cells exit the cell cycle and detach from the capsule in the lens transition zone [2,11] (Figure 1). The resulting nascent lens fiber cells subsequently initiate a maturation program characterized by cellular elongation [2], loss of organelles to form the lens organelle-free zone (OFZ) [1,12], and the abundant expression of crystallin proteins that reach concentrations as high as 450 mg/mL [13]. Key to the process of lens differentiation are a wide range of cellular remodeling events, including the elimination of organelles during OFZ formation. Although a number of proteolytic and other enzymatic processes have been demonstrated to be essential for lens fiber cell formation and the formation of the lens OFZ, the full range of pathways and components required for lens fiber cell remodeling and maturation remain to be identified. Recent data have provided evidence that autophagy is a critical requirement for lens fiber cell remodeling, organelle degradation, and transparency. This review summarizes our current knowledge of the role of autophagy in lens fiber cell differentiation, organelle elimination, and transparency.

## 2. Autophagy Overview

Autophagy is a cellular process that degrades and/or recycles damaged or excess cellular components. This process is particularly important for the development and homeostasis of tissues requiring high turnover or removal of cellular components, such as lens cells that undergo remodeling and organelle degradation during their differentiation program. Autophagy can be divided into three main types: macroautophagy, microautophagy, and chaperone-mediated autophagy [14,15]. During macroautophagy (hereafter termed autophagy), the target component is sequestered into a newly formed double-membrane structure called an isolation membrane or phagophore, which matures into an autophagosome. Autophagosomes ultimately fuse with lysosomes to form autolysosomes, wherein cellular components are degraded by resident acid hydrolases, creating molecular building blocks for recycling [16]. By contrast, in microautophagy, cytosolic material is directly engulfed by lysosomes [17]. Finally, in chaperone-mediated autophagy, specific proteins containing key recognition motifs are unfolded by cytosolic chaperones and translocated across the lysosomal membrane by the lysosomal-associated membrane protein 2A (LAMP2A) [18]. Autophagy can be further divided into the selective and non-selective forms. In the non-selective form, cellular components are randomly engulfed by autophagosomes, usually in response to stress or starvation conditions [19]. In selective forms of autophagy, specific organelles or molecules are targeted by autophagy receptors. The selective forms of autophagy include mitophagy (mitochondria), ER-phagy (endoplasmic reticulum), aggrephagy (protein aggregates), ribophagy (ribosomes), and xenophagy (foreign pathogens) [20].

While autophagy occurs at a basal level in all cells, it can be upregulated in response to cellular stress, such as nutrient or energy deprivation [21,22]. However, autophagy can also be upregulated if cells are required to carry out large-scale remodeling during development or differentiation, such as that required for lens fiber cell formation [23]. Impaired or deficient autophagy has been linked with multiple diseases, including cancer and neurodegenerative disease as well as metabolic disease and aging [24]. Broadly, the classic autophagy pathway can be divided into the following steps: initiation and nucleation of the phagophore, elongation and closure of the autophagosome, fusion with lysosomes, and finally, degradation of the cargo. These steps will be described briefly here, although they are discussed in detail in a number of excellent reviews [14,15,22,25,26,27,28]. The induction of autophagy is controlled by the activity of the mechanistic target of rapamycin (MTOR). Active mTOR inhibits the phosphorylation of UNC51-like kinase (ULK1) in the initiation complex consisting of ULK1 and its substrates ATG13 and RB1CC1/FIP200. When autophagy is induced, the ULK1 serine/threonine kinase is activated via autophosphorylation, binding ATG13, which bridges it to the scaffolding protein RB1CC1/FIP200, recruiting this induction complex to the forming phagophore [25]. The source of the membrane for the forming phagophore has been proposed to be plasma membrane or mitochondria, depending on the cell type and/or stress studied. However, several studies suggest that structures called omegasomes, which arise from the ER through the transfer of lipid membranes by WD-repeat proteins interacting with phosphoinositides (WIPIs) and ATG2, are the main source of phagophore in mammals [29,30]. Induction is followed by nucleation, which involves the recruitment of the ATG14-containing class III phosphatidylinositol 3-kinase (PtdIns3K) complex. This complex consists of PIK3C3/VPS34, PIK3R4/p150, and BECN1 (Vps30). Regulation of this complex occurs through proteins that interact with Beclin1 (BECN1), such as BCL2, which binds BECN1 and prevents its interaction with PIK3C3 or positive regulators such as AMBRA1 [25,26,27]. The elongation and closure of the phagophore involve conjugation systems consisting of ubiquitin-like proteins, namely the ATG12–ATG5-ATG16L1 complex and the well-studied ATG8/LC3 proteins. The ATG8 family proteins are divided into two subgroups: the LC3 (light chain 3) subfamily, which includes LC3A, LC3B, LC3B2, and LC3C, and the g-aminobutyric acid receptor-associated protein (GABARAP) subfamily, which includes GABARAP, GABARAPL1, and GABARAPL2 [25,26,31,32]. LC3B is commonly studied, and it is accepted as a marker of autophagosome formation in the classic autophagy pathway [33]. The ATG12–ATG5-ATG16L1 complex associates with the phagophore and dissociates once the autophagosome has been formed. LC3 is cleaved at its c-terminus by ATG4, yielding a cytosolic form. LC3-I. LC3-I is then conjugated with phosphatidylethanolamine (PE), producing LC3-II, which is embedded into the autophagosomal membrane [25,26,28]. Lipids are supplied to the expanding autophagosomal membrane via a combination of ATG2 and ATG9, two intermembrane and interleaflet lipid transporters [34,35,36,37]. Recent structural data show that ATG2 is a rod-like molecule with a hydrophobic tunnel that transports lipids, while ATG9 is a scramblase that redistributes those lipids in the autophagosome membrane [34]. In addition to ubiquitination systems, PI3P-binding ATG proteins (ATG18 and ATG21) and members of the WD-repeat protein that interact with the phosphoinositides (WIPI) family are required for the closure of the autophagosome [38]. Completely mature autophagosomes fuse with lysosomes in a process that involves the tether and SNARE proteins [26,28]. Here, the cargo is degraded and its components are recycled. In 2009, an ATG5/ATG7-independent autophagy pathway was discovered by Nishidia et al. [39], demonstrating an alternative to the classic autophagy pathway described above. In the alternative pathway, autophagosomes are generated in a Rab9-dependent manner through the fusion of isolation membranes with vesicles derived from the trans-Golgi and late endosomes [39]. Studies in the lens, as detailed in this review, have identified the involvement of selective and non-selective autophagy in both lens homeostasis and key elements of the differentiation process. These studies suggest that both the classic and alternative autophagy pathways are operating contemporaneously in the lens, indicating a redundancy between the pathways and highlighting the critical need for autophagy in lens homeostasis and differentiation.

## 3. Signaling Regulators Control the Induction of Autophagy to Form the OFZ

The process of eliminating lens organelles begins with the differentiating fiber cells in the center of the lens and expands outwards toward the lens equator [40], with a distinct border maintained between the developing lens organelle-free zone (OFZ) and the surrounding nascent fiber cells that still retain their organelles (Figure 1). Studies of the developing chick embryo lens provided some of the earliest evidence that the removal of lens organelles to form the OFZ involved an autophagic mechanism [41,42]. This was followed by studies showing that the removal of fiber cell organelles, including their mitochondria, ER, and Golgi, is achieved through the induction of autophagy-promoting signaling pathways [41,43,44]. It was also found that the signaling events that induce the removal of the lens fiber cell nuclei involve autophagic pathways. Nuclear elimination is a more complex process than the removal of non-nuclear organelles. It requires the regulation of multiple signaling pathways [43], ensuring that the end stage of the OFZ formation is accomplished without inducing fiber cell death [44]. In the mature lens, after the organelles are eliminated, homeostasis is maintained by factors supplied through cell–cell junctions [45,46] and the lens microcirculation system [47,48].

As the lens develops, suppression of the signaling effectors in the center of the lens that induce autophagy occurs coordinately with the removal of the lens organelles [41,43]. These include both the MAPK JNK [41] and PI3K [43] pathways and their downstream effectors Jun [41], Akt [43], mTOR, raptor, and p70S6K [41,43]. Their suppression induces molecules essential to the autophagy process, such as LC3B-II and BECN1, which localize to the autophagic vesicles in the central region of the lens where the lens organelles are removed [41,43]. BECN1 functions to promote the assembly of the phagophore membrane and participates in the initiating events in the autophagy pathway, while LCB3-II has key functions in the expansion of phagophore membranes, directing cargo to the phagophore, and the fusion of phagophores to form autophagosomes [33]. Importantly, both of these autophagy proteins localize specifically to the autophagic vesicles within which the lens organelles are being eliminated [41]. Together with electron microscopy analyses, these immunolocalization studies provided strong evidence that the non-nuclear organelles of the lens are removed in the autophagic vesicles to form the OFZ, as induced by the autophagy-signaling pathways [41]. In contrast to the requirement for the inhibition of Class I PI3K signaling for the induction of lens organelle elimination, it was reported that the Class III PI3K Pik3c3 does not have a role in mediating lens organelle loss [49].

The best studied autophagy signaling pathway is the “self-eating” process associated with the cellular response to stress, such as occurs in nutrient starvation and hypoxia and involves the suppression of the PI3K/Akt/mTOR/p70S6K signaling axis [50]. This pathway leads to the formation of autophagosomes that digest organelles to supply cells in a stress environment with the nutrients required to maintain/restore their homeostasis. It is the Class 1 family PI3Ks that are directly involved in the pathways that regulate autophagy [51]. They consist of both catalytic (p110α-δ) and regulatory (p85, p101) subunits, with Akt and Rac serving as their major downstream effectors [52]. The earliest evidence that the suppression of PI3K signaling is linked to lens OFZ formation is found in studies with a primary differentiating lens epithelial cell culture system that forms organoid structures called lentoids [53]. While inhibiting PI3K signaling has no impact on primary undifferentiated lens epithelial cells, blocking PI3K signaling after lentoid formation induces the elimination of the nuclei in these organoid structures, the hallmark of lens OFZ formation [54]. Years later, suppression of the canonical autophagy-inducing PI3K/Akt/p70S6K signaling axis was definitively linked to OFZ formation in the developing lens [43]. Both this PI3K/Akt signaling axis and the MAP kinase JNK signaling pathway were found to play essential roles in regulating the temporal removal of the mitochondria, ER, and Golgi from the central lens fiber cells to form the OFZ during development. These signaling pathways function through their impact on the mTORC1 complex proteins and the activation state (phosphorylation) of p70S6K [41,43]. The role of JNK in regulating the phosphorylation state of the mTORC1 complex proteins mTOR and raptor to induce autophagy was first discovered in these studies of lens development [41].

Functional studies performed ex vivo with isolated lenses placed in organ culture prior to OFZ formation provided direct evidence that the inhibition of both the JNK- and PI3K-dependent signaling pathways provides the mechanism for the induction of autophagy to removal lens organelles [41,43]. The inhibition of JNK signaling blocked the phosphorylation of the mTORC1 complex proteins mTOR and raptor and their downstream target p70S6K in lens fiber cells, initiating autophagy through the induction of the autophagy molecules ULK1 and Beclin and the conversion of LC3B-I into LC3B-II [41]. This resulted in the premature removal of the mitochondria, ER, and Golgi in the LCB3+ autophagic vesicles from the fiber cells in the center of the lens and the condensation and elimination of the central fiber cell nuclei [41]. Similar results were achieved by exposing organ cultured lenses to rapamycin [41], a drug that blocks the activation of mTOR to induce autophagy [55].

Inhibiting PI3K activity in lens organ cultures blocks the activation of Akt and the PI3K/Akt/mTORC1 complex effector p70S6K in lens fiber cells, signaling the conversion of LC3B-I into LC3B-II [43], which are all elements of the classical autophagy-induction pathway [33]. The exposure of lenses to pan-PI3K inhibitors that suppress the activation of all PI3K p100 catalytic subunits and their downstream effectors induces autophagy and the premature elimination of organelles, including the nuclei, from central lens fiber cells [43]. However, while the specific inhibition of the PI3K downstream effector Akt induces autophagy and the autophagy-dependent elimination of the lens mitochondria and ER, alone it is insufficient for the removal of lens nuclei [43]. These findings revealed that the suppression of another major PI3K target, such as Rac, is also required for nuclear elimination during the formation of the OFZ.

As lens fiber cells are the only cell type in which the nuclei are removed and long-term survival maintained, it is not surprising that the removal of their nuclei is a complex, multistep, and highly regulated process that protects them as their nuclear material is eliminated. It is expected that autophagy is required during lens nuclear elimination for the removal of nuclear breakdown products. Autophagic mechanisms involve the fusion of autophagic vesicles with lysosomes that contain the acid hydrolases that digest proteins, lipids, carbohydrates, and nucleic acids [56]. Similar to autophagosomes, LAMP+ lysosomes form in the lens fiber cells in a differentiation-state-specific manner [43]. Consistent with their function in the premature elimination of lens organelles, the LAMP protein and the formation of LAMP+ lysosomal vesicles are induced by the exposure of lenses to PI3K pathway inhibitors [43]. Another related finding is the link between phospholipase A/acyltransferases (PLAATs) [57] and the elimination of lens organelles [58]. The translocation of PLATT enzymes to the phospholipid membranes of mitochondria causes the deformation of the mitochondrial surface and the subsequent loss of mitochondrial membrane potential [59]. While depolarization of the mitochondria is not induced when PI3K activity is suppressed [43], the mitochondria become depolarized during lens development just prior to the time that they are delivered to the autophagosomes for elimination [60]. These findings suggest that PLAATs are likely to promote mitochondrial membrane depolarization during lens fiber cell differentiation, which drives the damaged mitochondria to the autophagic vesicles for their elimination.

Phosphoinositide-3-kinase-interacting protein 1 (PIK3IP1) is a membrane-linked p85-like subunit that binds to the p110 catalytic subunits of PI3K, blocking their interaction with the p85 regulatory subunit and preventing activation of the p110 catalytic subunit [61]. RNAseq studies revealed that this novel and little-studied negative regulator of PI3K signaling is highly upregulated in lens fiber cells, where it could function as the endogenous inhibitor of PI3K signaling to induce the autophagy-dependent formation of the OFZ [43]. In the developing lens, PIK3IP1 associates with the p110α, p110β, and p110γ catalytic subunits of PI3K specifically in the lens fiber cells at developmental times when autophagy is induced to remove the lens organelles [43]. This discovery provides the first evidence that PIK3IP1 is an endogenous regulator of PI3K signaling in the developing lens likely responsible for inducing the autophagy-dependent spatiotemporal removal of organelles to form the OFZ.

## 4. Autophagy Mechanisms in Non-Nuclear Organelle Degradation

A long-unanswered question in lens biology concerns how lens fiber cells eliminate their organelles to form the lens OFZ. Only three cell types remove all of their organelles in the absence of a specific stress and as part of their differentiation process: reticulocytes, keratinocytes, and lens fiber cells. In contrast to lens fiber cells that live as long as the lens itself, reticulocytes mature to become short-lived erythrocytes with a life span of 120 days, while keratinocytes terminally differentiate into corneocytes at the upper layer of the epidermis and are ultimately shed.

The mechanism of mitochondrial removal in reticulocytes has provided clues as to how this might be achieved during lens differentiation. A number of studies demonstrated that the selective autophagy pathway, namely mitophagy, was responsible for mitochondrial elimination in reticulocytes [62,63,64,65]. When Costello et al. [42] discovered mitochondria in autophagic vesicles throughout the differentiating fiber cells of the chick lens, this suggested a similar role for mitophagy in the elimination of mitochondria in the lens [42]. Mitophagy is the process whereby damaged or excess mitochondria are removed and their contents recycled for quality and quantity control [19]. Mitophagy is mediated by a number of receptors, some of which are targeted to the outer mitochondrial membrane, such as PARKIN, BNIP3, BNIP3L(Nix), FUNDC1, BCL2L13, FKBP8, and ATG32 [19,66]. Briefly, these receptors target mitochondria so that they are recognized by the forming autophagosomes, and the engulfed mitochondria are subsequently delivered to the lysosomes for degradation. Of particular relevance to the lens is the mitophagy protein BCL2 interacting protein 3-like BNIP3L(Nix), which has previously been shown to be essential for the removal of mitochondria in reticulocytes [62,63,64,67,68]. BNIP3L contains an Atg8-family protein-interacting region/LC3-interacting region (AIM/LIR) domain in its cytosolic n-terminus that binds Atg8/LC3 proteins to target the mitochondria to the autophagy machinery [68]. It forms a stable homodimer via its C-terminal transmembrane domain that inserts into the mitochondrial outer mitochondrial membrane. Recent studies have shown that dimerization is regulated by phosphorylation, and it is critical for the interaction with the Atg8 proteins and the recruitment of autophagy vesicles for mitochondrial elimination [69].

Examination of the spatial distribution of the BNIP3L transcript and protein in both chick [70] and mouse lenses [71] showed that both were higher in lens fiber cells in which mitochondrial elimination was taking place. The lenses of a global BNIP3L knockout mouse retained mitochondria at the center of the P1 and P14 lenses, demonstrating a requirement for BNIP3L in their elimination [71]. Interestingly, examination of BNIP3L knockout mouse lenses for levels of both the endoplasmic reticulum and Golgi apparatus showed that these two organelles were also retained in the center of the lens [71]. Although previous work had localized BNIP3L to the ER in non-lens cells [72], a role in ER quality or quantity control had not been established in these studies, and no other study had localized BNIP3L to the Golgi apparatus, demonstrating a novel role for BNIP3L. The findings in the BNIP3L knockout mice suggested that BNIP3L was responsible for the elimination of three non-nuclear organelles to form the OFZ of the lens.

Previous work in a number of cell types demonstrated that the master regulator of the hypoxic response, hypoxia-inducible transcription factor a (HIF1a), regulated hypoxia-induced autophagy by upregulating the expression of BNIP3L [73]. Under normoxic conditions (21% O_2_), the HIF1a protein is targeted for degradation by members of the 2-oxoglutarate-dependent dioxygenase superfamily of prolyl hydroxylases (PHD1, PHD2, and PHD3) through the hydroxylation of the proline resides in its oxygen-dependent domain [74,75]. Hydroxylated HIF1a associates with the von Hippel–Lindau tumor suppressor protein that targets HIF1a for rapid proteasomal degradation [74,75]. A further method of regulation involves the hydroxylation of an asparaginyl moiety in the c-terminus domain by factor-inhibiting HIF (FIH). This hydroxylation reduces the HIF1a transcriptional activity by disrupting its association with the important co-factors p300 and CREB-binding protein (CBP) [74,75,76]. Both PHDs and FIH are oxygen-dependent enzymes; therefore, in reduced oxygen conditions, the HIF1a protein is no longer targeted for degradation. Instead, it translocates to the nucleus, where it dimerizes with constitutively expressed HIF1b (ARNT) and other co-factors to regulate the expression of the genes required for the hypoxic response [74,75,76].

Consistent with a role for HIF1a in BNIP3L expression in the lens, the lens is one of very few tissues to reside in a hypoxic environment. During development, the fetal vasculature made up of the tunica vasculosa lentis and the anterior pupillary membrane feeds the lens [77]; however, shortly before birth, the vasculature regresses, leaving the lens without a direct oxygen supply [78]. The lens, therefore, relies on the diffusion of oxygen across the cornea and through the aqueous humor. In addition, the high number of active mitochondria in the anterior epithelial layer consume much of the surface oxygen [79,80,81,82]. Measurements of oxygen levels in both human and bovine lenses confirm that the oxygen level at the surface of the lens is around 2% O_2_ and that the lens contains an oxygen gradient with an almost 20% drop in oxygen levels from the surface to the core [80,81]. This gradient creates a hypoxic microenvironment in the region of the lens, the transition zone, where organelle elimination is being initiated (Figure 1). Consistent with this, incubation of embryonic chick lenses in hypoxic or low oxygen conditions (1% O_2_) accelerated the degradation of non-nuclear organelles relative to lenses in normoxic conditions (21% O_2_) [83]. In addition, exposure of embryonic chick lenses to an antagonist of the PHD cofactor α-ketoglutarate, dimethyloxalylglycine (DMOG), under normoxic conditions stabilized the HIF1a protein, increased the BNIP3L transcript and protein levels, and enhanced the degradation of non-nuclear organelles [83]. By contrast, inhibition of HIF1a transcriptional activity under hypoxic conditions (1% O_2_) in the same chick lens model using the chemical inhibitor Chetomin decreased the transcript and protein levels of BNIP3L and reduced the elimination of non-nuclear organelles relative to degradation in lenses incubated in hypoxia alone [83]. ChIP-qPCR confirmed that HIF1a binds to the 5′ untranslated region of the BNIP3L gene following the treatment of embryonic chick lenses with the HIF1a activator DMOG [83]. Collectively, the evidence suggests that the low oxygen environment of the lens promotes HIF1a-induced autophagy via the induction of BNIP3L expression. Combined with the presence of large numbers of autophagosomes containing remnants of mitochondria, the most easily identifiable organelle during degradation, these data suggest that hypoxia-induced autophagy is key to organelle elimination in the lens forming the lens OFZ and to continued organelle degradation during lens differentiation.

## 5. Lens Autophagy Structures

Autophagy is a conserved catabolic process in which a double-membrane phagophore encloses cellular material that is destined for degradation via fusion with a lysosome containing hydrolytic enzymes. In the original description of autophagy in liver by Dr. C. de Duve in 1965 [84,85], autophagic vesicles with heterogenous contents were observed with their double-membrane covering using the thin section electron microscopy technique, which has become the gold standard for identifying autophagic vesicles and contributed to the characterization of numerous other cellular organelles and processes, leading to the Nobel Prize for de Duve, Claude, and Palade in 1974. The primary contribution to this 1974 Nobel Prize by Dr. de Duve was the discovery of the lysosome and peroxisome, and about four decades later in 2016, Dr. Y. Ohsumi was awarded the Nobel Prize for the molecular description of the autophagic process. The discoveries of the 1974 laureates formed the basis for modern cell biology, and their approaches highlighting the value of morphology are still important today, allowing us to describe a new organelle, the nuclear excisosome, in chick embryo lenses that degrades the nuclear envelope toward forming the organelle-free zone (OFZ) [86]. More recently, we described the nuclear excisosome in the prosimian Galago (bush baby) monkey lens, where the cellular structures are distinct from those in the chick embryo lens but carry out essentially the same function [87]. Presented here are several examples of autophagic vesicles in lenses from different species and recent observations on autophagy and the nuclear excisosome in the Galago (bush baby) monkey model.

**Figure 2:**
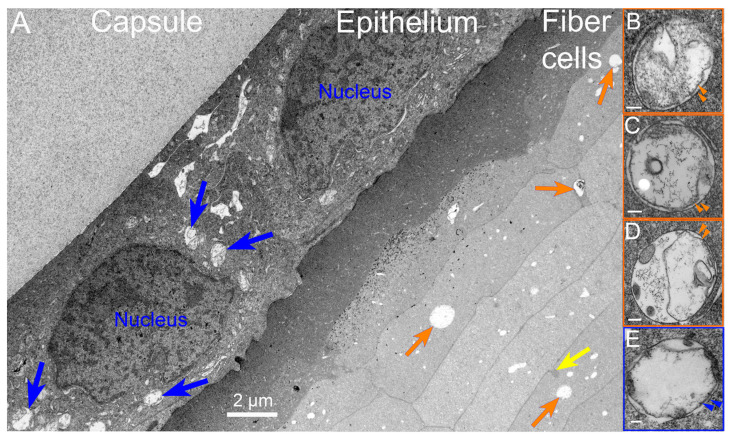
Low-magnification transmission electron microscopy (TEM) overview of the outer cortex of an adult macaque monkey lens. (**A**) This image, taken near the equatorial plane, displays the capsule, which is nearly homogeneous (about 10 µm thick), and the epithelium (about 7 µm thick), which has a very smooth interface with the capsule. The epithelium contains two prominent nuclei and numerous organelles, including autophagic vesicles (blue arrows) showing the classic double-membrane profile and heterogeneous contents. A narrow band of elongating fiber cells is generally more darkly stained, with the cells having irregular cross-sections that blend into classical fiber cells, which form radial cell columns and have flattened hexagonal shapes (about 2 × 10 µm). Several autophagic vesicles are labeled (orange arrows), and one ball-and-socket interdigitation (yellow arrow) typical of this region is about 0.5 µm long. (**B**–**E**) Insets are examples of autophagosomes from different regions in the same sample to illustrate the double-membrane structures (double arrowheads) at high magnification. The scale bars in the insets are 100 nm. (**B**) Autophagic vesicle shows degradation of cytoplasmic material in the space between two large irregular vesicles. A small segment of ER appears just outside the autophagic vesicle (upper left). (**C**) Extensive protein degradation in the center leaves a typical reticulated network. A vesicular structure with three thin lipid bilayers is darkly stained and next to a low-density vesicle-like circular profile, probably representing material lost during processing, which illustrates that the uniform background staining in all the examples is preserved fluid after degradation by hydrolytic enzymes. A segment of ER is preserved (upper right). (**D**) A variety of vesicular structures are being degraded along with protein, leaving reticulated patterns. A segment of ER is preserved (lower right). (**E**) Degradation has proceeded in this epithelial autophagic vesicle interior, leaving a few fragments extending into the preserved fluid.

**Figure 3:**
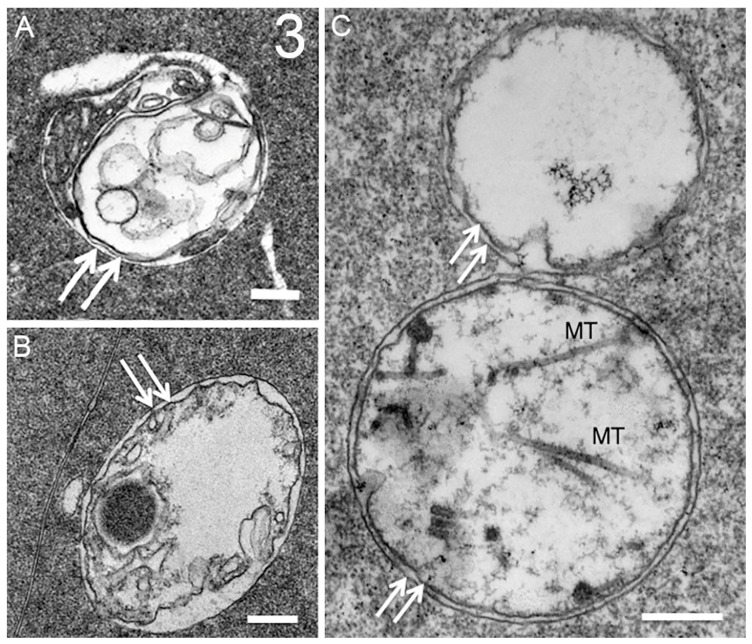
Similar double-membrane autophagosomes are found in lens fiber cells from a variety of diverse species. (**A**) Heterogeneous contents include an elongated structure (upper left), which has internal membranes that are probably partially degraded mitochondrial cristae. Adult human donor lens. (**B**) Heterogeneous contents include a dark, circular, and uniformly stained object that is similar to the core of MLBs (multilamellar bodies). Chick embryo D15 lens. (**C**) Lower double-membrane vesicle displays several structures that are most likely microtubule segments (MT, linear 25 nm diameter tubules), which could only occur within an autophagic vesicle. Adult rabbit lens. Scale bars are 250 nm. Double-membrane cover is indicated by arrows in all four examples.

Discounting some uncertainty in the literature about the presence of autophagy in the lens, we have been able to identify numerous autophagic vesicles in the epithelium and developing fiber cells from several species, initially in adult human lenses and chick embryo lenses [42]. In this first morphological study of lens autophagy, we emphasized the heterogeneous contents of the autophagic vesicles, some of which was consistent with the mitochondria or multilamellar lipid. Such lipid arrays with 5 nm spacing (indicating a minimal protein content) were proposed to be conserved and recycled, and their appearance is a common feature of the autophagy processes in the lenses examined [42]. The vesicle coverings were mainly single membranes, indicating that the degradation had proceeded to the autolysosome stage, although there was no doubt about their identification as autophagic vesicles. Around the same time, a study of modified mouse lenses was published that clearly showed the double-membrane profile of the autophagic vesicles [88], which was subsequently confirmed in chick embryo lenses a year later [41]. To emphasize the typical appearance of the autophagic vesicles in the vertebrate lens, the results concerning macaque monkey lenses illustrate the appearance and distribution of the autophagic vesicles (Figure 2). Note the micron-sized autophagic vesicles at low magnification in the epithelium and young fiber cells. Nearly circular and low density due to degradation, they are numerous up to the OFZ, supporting the proposal that they are involved in OFZ formation. At high magnification in the insets, the heterogenous contents and double-membrane profiles are pronounced in these examples of autophagosomes. Similar preservations of autophagosomes in a variety of species are presented (Figure 3) and show features of the original objects being degraded. Although whole mitochondria are easily recognizable, fragments still maintain the unique features of the envelope or cristae (Figure 3A). The high density and circular profile shown in Figure 3B can indicate the source being condensed protein, as found in multilamellar bodies [89]. A 25 nm diameter tubular object, consistent with a microtubule commonly found free in the cytoplasm, when occasionally captured in segments in an autophagic vesicle (Figure 3C) is representative of the degradative and recycling processes.

**Figure 4:**
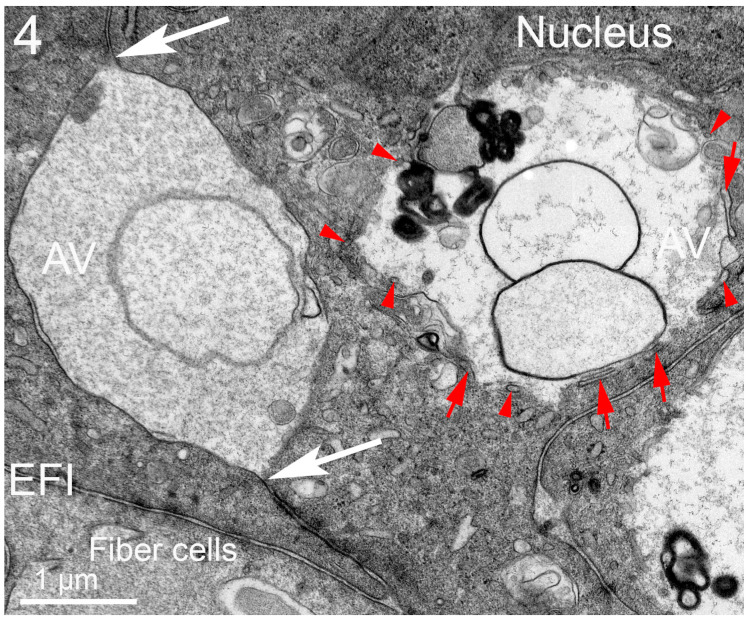
Extensive autophagic activity in the equatorial epithelium of prosimian Galago (bush baby) monkey lenses. A large autophagosome (AV) is observed in contact with a nucleus (upper right), which probably signals the supply of new ER membrane for expansion. The double-membrane surface is modified to present interconnected tubules with two membranes visible (red arrows) and cross-sections that are round or oval (red arrowheads). These membrane processes form an open meshwork that surrounds the autophagic vesicle, allowing hydrolytic enzymes to enter and degradation products to exit, including here from the degradation of proteins, large vesicles, and pure lipid aggregates (densely stained clusters with 5 nm spacing visible at higher magnification). This is an example of a classic autophagic vesicle. By contrast, the large autophagic vesicle on the left (AV) has a different structure and origin. Note that the large internal space is surrounded by a single membrane that pinches off at the borders (white arrows), suggesting that the space is equivalent to the extracellular space between the adjacent cells and that the membrane covering is derived from plasma membranes, not the ER or another internal organelle. In this initial form, the extracellular space structure should more appropriately be called a vacuole rather than a vesicle. The heterogeneous contents are in the process of degradation but, in this case, most likely arrive through cellular secretion, as do the hydrolytic enzymes causing the degradation. This is an example of a new type of autophagic vesicle that significantly increases autophagy in this epithelium near the lens equator. The unique origin can be confirmed by following the double membrane from the lower sealed point (lower white arrow) to the interface with the fiber cells at the epithelial–fiber cell interface (EFI), where the punctate adherens junctions along the EFI extend into the epithelium.

The epithelium near the equator of Galago (bush baby) monkey lenses is specialized for the production of nuclear excisosomes and for the extensive autophagy supporting the synthetic activity. The large autophagic vesicles are clearly of two distinct variations (Figure 4). The autophagic vesicle close to a nucleus (on the right) is classic in that it appears to originate from the outer nuclear envelope, which is also the beginning of the ER. It contains a variety of degrading proteins, membrane vesicles, and multilamellar lipid arrays. The outer membrane is modified from the initial phagophore double membrane. The paired closely associated membranes appear to partially fuse into tubules, which, when cut longitudinally, appear as double membranes (Figure 4, long red arrows) and, when cut cross-sectionally, appear as small circular profiles (Figure 4, red arrowheads), giving the overall appearance of a scalloped perimeter. This arrangement may provide a porous meshwork covering for the autophagic vesicle, allowing hydrolytic enzymes to enter and degraded products to exit. An autophagic vesicle of nearly the same size (Figure 4, left) shows the expected degradation of circular vesicles and protein but is formed by the membranes of adjacent epithelial cells by enlarging the extracellular space between the cells and sealing the ends (Figure 3, white arrows). The material within the extracellular space is most likely generated locally based on the lack of material entering from the capsule into the outer layers of the epithelium and the large size of the extracellular space compartments. The area in sections devoted to these and classic autophagy vesicles is nearly equal to the area of the nucleus, indicating enhanced autophagic activity. The morphological data, therefore, support the hypothesis that the material in the cytoplasm enters the extracellular space via exocytosis along with hydrolytic enzymes to degrade the contents.

**Figure 5:**
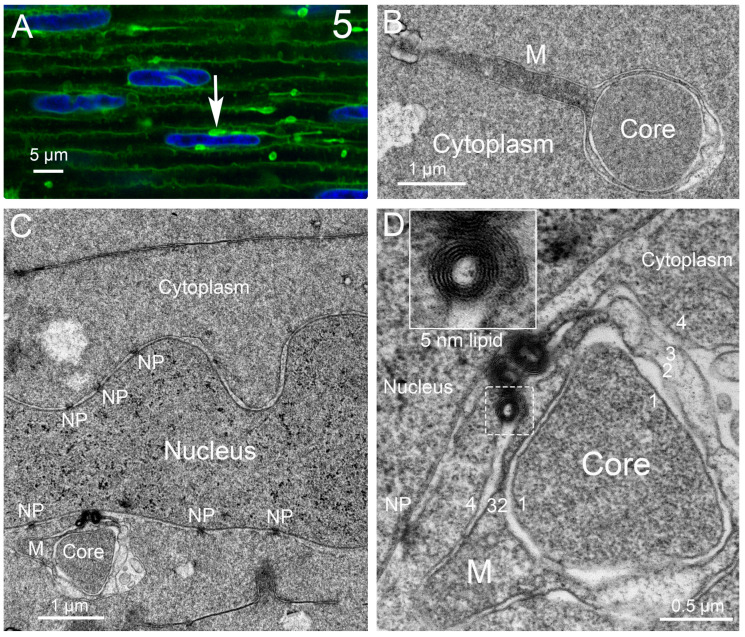
Nuclear excisosomes in a Galago (bush baby) monkey lens. (**A**) Confocal imaging (Zeiss 880 Airyscan super-resolution) shows the nuclei near the OFZ being degraded by nuclear excisosomes (NE). Lipid-rich globular structures (arrow) and thin linear strands are labeled with the membrane dye, DiI. (**B**) TEM of an NE strand cut in a cross-section with a core surrounded by four membranes and a large tail of the mitochondrion, which gave rise to the structure in the equatorial epithelium. The NE is free in the cytoplasm and remains so until it contacts a nucleus. (**C**) Binding of an NE with an attached mitochondrion (M) to a nuclear envelope to initiate degradation. Six nuclear pores are indicated (NP). (**D**) Degradation has begun based on the release of 5 nm multilayer aggregates of lipids (boxed region and inset). The core is surrounded by a single membrane (numbered 1), and outside the core is the pair of cristae membranes (numbered 2, 3) connected to the mitochondrion (M) and most likely releasing the enzymes needed for the degradation of the nuclear envelope. The outer membrane (numbered 4) is visible in several locations, including connecting to the covering of the mitochondrion.

Recent literature suggests that the membranes contributing to autophagic vesicles may be diverse, including organelles other than the ER as well as the plasma membrane [90]. This is the first example of the space between the epithelial cells being converted into an autophagic vesicle in the lens or any tissue. The production of the nuclear excisosome in the Galago (bush baby) lenses is extensive based on the number of bead-on-a-string structures visible in the confocal images (Figure 5A) [87]. The active unit is the unique four-membrane structure mainly contained in the narrow string or rod-like structure to the right of the contact region with the nucleus (Figure 5A, arrow) [87]. The cross-section of the rod gives the four-membrane profile with the dark core (Figure 5B), which has a pronounced mitochondrial tail from the mitochondrion responsible for its formation in the epithelium. This complex then moves around the fulcrum and enters the cytoplasm of a young fiber cell, where it will eventually migrate to a nucleus to be degraded, such as shown for a different four-membrane complex (Figure 5C). At high magnification, the core is surrounded by a single membrane (membrane 1), and it is adjacent to a pair of cristae membranes (membranes 2 and 3). On the left is an attached mitochondrion (M) that appears to be contributing its cristae and is covered by a membrane (membrane 4) that has fused with the outer nuclear envelope membrane to initiate degradation, as evidenced by the dense cluster of pure lipid multilayers (with 5 nm spacing, inset). The images support the hypothesis that the nuclear excisosome releases enzymes from the modified cristae (membranes 2 and 3) to break down the nuclear envelope membranes and release lipids analogous to the nuclear excisosomes described previously that do not display the attached mitochondria [87]. It is common to observe the four-membrane structure with an attached mitochondrion and rare to see a free mitochondrion in the fiber cell cytoplasm (although many are seen in the epithelium), suggesting that the energy for the autophagic and nuclear excisosome-controlled degradation processes is provided by the attached mitochondria.

## 6. Evidence against Autophagy in Organelle Elimination

Despite the numerous studies detailed in Section 3, Section 4 and Section 5 demonstrating the roles of autophagy and mitophagy in lens organelle degradation to form the OFZ, evidence to the contrary does exist. The recent analysis of the OFZ formation of in vivo lenses from zebrafish and mice report that the PLAAT1 and PLAAT3 enzymes, respectively, cause membrane damage and loss of membranous organelles without involving autophagy [58]. Alterations in the membrane structure documented in electron micrographs included rupture of the outer mitochondrial membrane, leaving exposed edges, and internal swelling and disruption of cristae [58]. These observations were supported by a study using HeLa cells in a culture treated with PLAAT3, which showed similar damage to the mitochondria, endoplasmic reticulum, and other organelles [59]. Such distinctive changes to the membranes were not observed in chick embryo or adult Galago (bush baby) lenses where high-resolution electron micrographs were available from the epithelium through the OFZ [86,87]. For example, Figure 5B in this review displays a nuclear excisosome with an attached mitochondrion that has an intact and smooth membrane around the mitochondria and the four-membrane structure. Similarly, the nuclear excisosome attached to the degrading nucleus in Figure 5C and D shows a smooth uniform membrane around the mitochondria up to the point of contact with the outer nuclear envelope membrane. Previous studies in the embryonic chick lens show mitochondria in various states of degradation in the autolysosomes [42], suggesting membrane rupture is not part of the organelle degradation process, at least not in chick lens. The PLAAT studies support the hypothesis that the specific actions of PLAATs in the lens contribute to lens OFZ formation and that these functions may differ among species. However, studies on PLAATs alone do not rule out the possibility that a multitude of overlapping mechanisms are likely involved in coordinating the degradation of lens organelles.

One of the first studies to examine the role of autophagy in lens OFZ formation utilized an ATG5 knockout mouse [91]. The lenses of the ATG5^−/−^ mouse showed degradation of both the nuclei and endoplasmic reticulum 0.5 days post-birth. The authors also concluded in this study that autophagy is not required for organelle degradation in erythrocytes [91]; however, many studies have since shown that BNIP3L-mediated mitophagy is the key pathway for mitochondrial elimination in reticulocytes [62,63,64,67,68]. This data suggests that while an ATG5 pathway is not required, autophagy/mitophagy could still play a role in organelle elimination in the lens in the same way as it does for erythrocytes. In a further study, the conditional knockout of ATG5 or PIK3C3 in mouse lenses also failed to prevent organelle degradation for OFZ formation [49]. This confirms the initial data showing that ATG5 is not alone required for OFZ formation. The lens-specific deletion of PIK3C3, which is involved in ATG/7-independent autophagy, also failed to prevent OFZ formation [49]. This raises the question of whether a single mediator such as BNIP3L could recruit autophagosomes to organelles targeted for degradation regardless of the pathway of initiation. As detailed in Section 4, BNIP3L knockout mouse lenses retain organelles [71], while BNIP3L contains an AIM/LIR domain that can recruit LC3B-labelled autophagosomes, although it has not been shown that BNIP3L interacts with the Rab9a-generated autophagosomes formed in the alternative pathway. It is also possible that there is some redundancy between the pathways and that the large-scale degradation of organelles uniquely required for lens OFZ formation may result in both pathways being highly active and called upon at the same time in the region of the lens where the organelles are actively being eliminated. A double knockout of both pathways could provide some evidence of this, although the complex mix of multifunctional proteins that are involved in autophagy means it is difficult to completely ablate the process through genetic modifications. Although non-classical autophagy pathways have not been identified in the lens, the lens indeed expresses all of the components required for alternative autophagy [70]. Further evidence comes from data from ATG5 KO mice, which suggests that an alternative pathway does function in the lens, since organelles are still degraded in the absence of ATG5 [49]. Mutations in the scaffolding protein FYCO1, which functions in the transport of autophagic vesicles along microtubules, result in autosomal recessive congenital cataracts [92]. The knockout of FYCO1 in mice was shown to result in decreased autophagosomes and retention of organelles in the lens [93]. In contrast, a separate FYCO1 knockout mouse showed normal degradation of the nuclei, mitochondria, and ER [94]. Additional evidence of autophagy involvement in organelle elimination for OFZ formation is provided by gold standard electron micrographs showing mitochondria contained within autolysosomes throughout the region of the lens where organelle degradation takes place [42]. To date, only mitochondria have been identified in autolysosomes, since other organelles have less distinguishable morphology in EM analysis. Curiously, a study by McWilliams et al. using a MitoQ reporter mouse found widespread autophagy but little mitophagy in mouse lenses [95]. This stands in contrast to studies by Basu et al., who showed the co-localization of the autophagosome marker LC3B and mitochondrial marker TOMM20 throughout embryonic chick lenses [41]. Although much of the evidence of autophagy-mediated organelle elimination discussed in Section 3, Section 4 and Section 5 is derived from ex vivo cultured lenses, EMs of lenses with no chemical manipulation suggest that it is an ongoing process during lens development and differentiation [42]. It is clear that the myriad proteins and pathways involved in autophagy/mitophagy make it a complex process to study; however, robust evidence suggests that it plays a role in organelle elimination.

## 7. Role of Autophagy in Cataractogenesis

**Figure 6:**
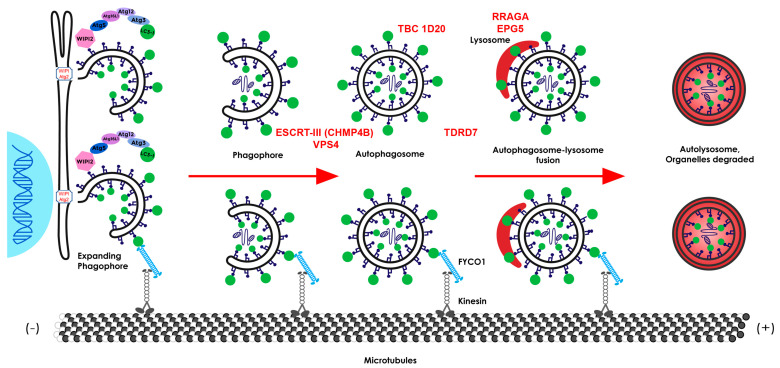
Overview of autophagy, with the components implicated in human cataractogenesis shown in red above the steps in which they are active (shown as red arrows). FYCO1 is shown below, binding to LC3 and PtdIns(3)P, which are active through the formation of the autolysosome.

Mutations in a number of autophagy-associated genes have been implicated in inherited cataracts (Figure 6). Early speculation that autophagy was linked to cataract arose when progressive posterior polar/subcapsular cataracts were associated with mutations in *CHMP4B* (charged multivesicular body protein 4b), a core subunit of the endosomal sorting complex required for transport-III (ESCRT-III) machinery, which is important for cellular membrane remodeling and scission processes, including, but not limited to, autophagy [96,97,98]. Germline knockout of the mouse *Chmp4b* gene (also known as *mSnf7-2*) was found to be embryonically lethal and the loss of *Chmp4b* expression caused autophagosome accumulation in cultured cortical neurons and fly eyes [99,100]. Heterologous overexpression studies suggested that a mutant form of *CHMP4B* (p.D129V), which is associated with human cataract, impaired the endosomal pathway [96] and also inhibited binding to chromatin, thereby implicating CHMP4B in the autophago-lysosomal degradation of micronuclei and other extra-nuclear chromatin [101]. In addition to early-onset forms of autosomal dominant cataract [96,102,103,104], *CHMP4B* variants have recently been associated with age-related nuclear cataract [105] (https://cat-map.wustl.edu, accessed on 29 November 2022). Whereas germline knock-in of the CHMP4B-(p.D129V) mutant did not elicit cataract in heterozygous mice, homozygous mice were embryonically lethal, and the conditional knockdown of CHMP4B in the lens resulted in severe inhibition of lens growth and fiber cell differentiation, rendering cataract modeling studies challenging [106]. While CHMP4B has been shown to participate in endolysosome membrane repair [107,108], endoplasmic reticulum (ER)-phagy [109], and phagophore closure during mitophagy [110], the exact pathogenic mechanism(s) underlying CHMP4B-related cataract remains unclear. Beyond autophagy defects, impaired cytokinetic abscission, which is associated with reduced CHMP4B accumulation at the intercellular bridges between PI3K-C2a deficient lens epithelial cells, has been shown to result in early lens senescence and cataract formation in fish, mice, and humans [111], suggesting that multiple pathogenic pathways may contribute to CHMP4B-related cataract formation.

Autosomal recessive congenital cataracts are also linked to mutations in the scaffolding protein FYCO1, which is important in the transport of autophagic vesicles by microtubules [92]. Since then, *FYCO1* mutations have been shown to be a relatively common cause of cataract, causing about 2.2% of reported independently ascertained families, although this can increase dramatically in some isolated populations, accounting for almost 14% of autosomal recessive cataracts in the Pakistani population [112] and 86% of inherited cataracts in the Yakut population of Eastern Siberia [113]. FYCO1 forms a complex with RAB7 and LC3 that mediates microtubule plus end-directed transport for selective autophagy [114,115]. The binding of FYCO1 and RAB7 is facilitated by NINL (NLP), which accelerates the formation of autophagosomes [116], and the phosphorylation of LC3B by STK4 decreases the FYCO1 binding and transport of autophagosomes [117]. LC3-FYCO1 binding occurs through the LIR sequence on FYCO1, and when the complex occurs, FYCO1 undergoes a conformational change that allows binding to PtdIns(3)P. The LIR in the FYCO1 extends over nine amino acids and is an F-type motif that appears to be specific to LCA [118,119]. FYCO1 also appears to be required for mTORC1 activation in concert with ZFYVE27 (protrudin) by relocating lysosomes containing mTOR to the plasma membrane [120]. The role of FYCO1 in inherited cataracts has been confirmed in mouse models [93,94]. Lenses from FYCO1 knockout mice show extensive vacuoles and large irregularly shaped fiber cells, while lens cells show increased expression of autophagy genes at the mRNA and protein levels but decreased autophagosomes and autophagic flux accompanied by the accumulation of p62 and retention of organelles, including the ER, Golgi apparatus, and mitochondria, in differentiating fiber cells [93]. While previous studies showed FYCO1 to be required for efficient basal autophagy [118], in the lens it appears to have a major impact on the elevated levels of autophagy required to eliminate organelles during lens fiber cell differentiation [93].

Mutations in RRAGA, a GTPase regulator of the mTORC1 pathway, have been associated with autosomal dominant congenital nuclear cataracts [121]. Mutations in RRAGA upregulated mTORC1 phosphorylation and thereby decreased autophagy. Mutations in *TDRD7*, a component of the RNA granules active in RNA processing and expressed in many tissues, have been shown to cause isolated hereditary cataracts as well as cataracts associated with glaucoma or azoospermia [122,123], and this gene has also been associated with age-related cataract [115,124]. The depletion of TDRD7 in mice and humans causes the failure of autophagosome fusion with lysosomes, resulting in autophagosome accumulation and disruption of autophagic flux [125]. In addition, several multisystem genetic syndromes featuring cataracts have been associated with other autophagy-related genes. Loss of function mutations in the gene responsible for TBC1D20 (TBC1 domain family, member 20) have been shown to cause autosomal recessive Warburg micro syndrome 4, which involves eye, brain, and endocrine abnormalities in humans, and male infertility with nuclear cataracts in blind-sterile mice [126,127]. TBC1D20 is a GTPase-activating protein that functions as a regulator of autophagosome maturation, which is consistent with an important role for autophagy in maintaining lens transparency. Homozygous or compound heterozygous mutations in the gene responsible for EPG5 (ectopic P-granules autophagy protein 5) have been found to underlie Vici syndrome, a rare autosomal recessive disorder involving agenesis of the corpus callosum, cataracts in about 76% of patients, cardiomyopathy, psychomotor delay, hypopigmentation, and combined immunodeficiency [128,129,130]. EPG5 has been implicated in autolysosome formation, and muscle biopsies and fibroblasts from Vici syndrome patients reveal a severe defect in autophagosomal clearance, resulting in the failed delivery of autophagic cargo to lysosomes. Recently, mutations in the ESCRT-III-associated protein VPS4A (vacuole protein sorting 4a) have been shown to cause an autosomal dominant syndrome characterized by severe neurodevelopmental delay, growth impairment, congenital dyserythropoietic anemia, and cataracts [131,132]. VPS4A is an ATPase that regulates the dynamics of ESCRT-III subunits, including CHMP4B, and expression studies of several human VPS4A mutants were consistent with dominant negative effects resulting in enlarged endosomes.

The deletion of Atg5 in mice has been associated with age-related cataracts and the deletion of Pik3C3 with congenital cataracts in mice [49], although neither deletion alone appeared to affect organelle degradation, and neither molecule has been associated with cataracts in humans. Unexpectedly, the ablation of Gja8b in zebrafish was found to decrease macroautophagy and cause cataracts, which could be relieved by the autophagy stimulator rapamycin [133]. Finally, autophagy-independent mechanisms for lens organelle degradation involving DNASE2B and the phospholipases in the phospholipase A/acyltransferase (PLAAT) family have been discovered [58]. However, it remains to be determined if DNASE2B and/or PLAAT gene variants are associated with cataracts.

## 8. Summary

The collective findings from multiple laboratories using chick and mouse model systems, non-human primates, and clinical studies in humans provide evidence that autophagy is essential for achieving mature lens structure, homeostasis, and transparency. The key autophagy structures, ranging from double-membrane autophagosomes to single-membrane autolysosomes, are found throughout the lens in both epithelia and fiber cells, which are involved in multiple tissue-remodeling and protein recycling events important for both lens epithelial cell homeostasis and lens fiber cell differentiation. Autophagy in the lens is coordinated by the precise regulation of the key signaling pathways that initiate autophagy to drive the critical steps in the lens differentiation program, such as the elimination of organelles to form the core lens organelle-free zone. The low oxygen environment of the lens drives HIF1a-induced autophagy via the upregulation of BNIP3L expression, resulting in the specific elimination of the mitochondria, endoplasmic reticulum, and Golgi apparatus during lens fiber cell differentiation. Finally, pioneering studies on the structural requirements for the elimination of nuclei during lens differentiation reveal the presence of an entirely novel structure associated with degrading lens nuclei, which is termed the nuclear excisosome. Thus, cataract resulting from the mutation of key autophagy proteins in both humans and animal models strongly suggests that autophagy is a requirement for lens differentiation and transparency, and both structural and mechanistic studies delineating the role of autophagy in the lens provide a theoretical basis for this.
